# Impact of climate change on the habitat range of monarch butterfly (*Danaus plexippus*)

**DOI:** 10.1038/s41598-025-17443-x

**Published:** 2025-09-23

**Authors:** Sanad H. Ragab, Michael G. Tyshenko, Marwa Waseem A. Halmy

**Affiliations:** 1 Department of Zoology and Entomology, Faculty of Science, Al-Azhar University, Nasr city, Cairo Egypt; 2 Risk Sciences International, Ottawa, Canada; 3 Department of Environmental Sciences, Faculty of Science, Alexandria University, Alexandria, 21511 Egypt

**Keywords:** Migratory insects, Ensemble model, Conservation, Global, Species distribution models, R packages, Ecology, Zoology, Ecology

## Abstract

*Danaus plexippus* L. (Linnaeus, 1758) (Lepidoptera: Nymphalidae) is well-known and captivating migratory insect that cannot overwinter in temperate climates. Although common throughout North America, monarch butterfly populations appear to be declining widely in their geographically distinct areas. Rapid climatic change is posing a serious threat to monarch butterfly populations, especially the migratory groups in the east. This study assesses how several climate change scenarios will affect the global distribution of monarch butterflies. Using climate projections from the Coupled Model Intercomparison Project Phase 6 model, we conducted projections for the near future (2021–2040) and far future (2041–2060, 2081–2100) under high (SSP5_8.5) and low (SSP1_2.6) emission scenarios. We estimated habitat gain, loss, and stability for *D. plexippus*. True Skill Statistic (TSS) and the Area Under the Curve (AUC) were used to assess the model’s performance. The results indicated that annual precipitation, land cover, and altitude (Alt) were the most influential factors affecting *D. plexippus* distribution. Potential habitat shifts were observed in Central Asia, Africa, Europe, and North America, with both gains and losses. These findings highlight the interconnected relationship between the climatic factors and distribution of migratory insects, emphasizing the need for targeted conservation efforts to mitigate climate change impacts on *D. plexippus* populations. Management strategies should prioritize habitat restoration, focus on overwintering site preservation, and implement adaptive management approaches. This research provides an evidence base for adaptive management to minimize climate change impacts on migratory insect populations.

## Introduction

Monarch butterflies (*Danaus plexippus*), are a fascinating and well-researched migratory insect species. A significant number of monarchs migrate twice a year. One generation of adult monarchs migrates southward from the northern United States and southern Canada in the fall to spend the winter in the central Mexican mountains^1^. Monarch butterflies are common through North America but appear to be experiencing wide-ranging declines in their geographically separate populations^2–4^. Monarch butterflies are present in various African countries, but there are no well-established overwintering sites analogous to Mexico or California. Cross-continental sightings are reported in countries such as South Africa, Kenya, and Tanzania. In Europe, monarch butterfly populations are primarily found in the Mediterranean region, where the climate is more favorable for their survival^5^. The eastern migratory populations of *Danaus plexippus*, face a significant threat because of the rapid impacts of climate changes^6,7^. Factors that influence the distribution of the species include numerous biotic and abiotic factors like climate change and the reduction of its main host plant, the milkweed^8,9^.

A change in breeding time with earlier spring breeding was detected in the butterfly populations of Mexico and southern United States, attributed to the rise in temperature and decreased host plant availability which was also recognized due to climate change^10^. Monarch butterfly recruitment rates are directly and indirectly impacted by changes in spring and summer weather, which is especially important to population dynamics^11^. Long, hot and dry temperatures shorten adult lifespans and limit their ability to reproduce, while prolonged wet and cool conditions cause shorter growth times and lower egg-laying rates^12^. The assessment of lethal and sublethal impacts of exposure to temperatures beyond the ecological tolerance revealed that long-term exposure to high daytime temperatures more than 36 °C increased larval mortality, resulted in developmental changes including reduced and slower pupation rates, and resulted eventually in smaller adult size^13^.

Some studies support the effects of climatic conditions on butterfly species distribution^14^ directly on the larval stages and indirectly by influencing milkweed, availability, the monarch’s preferred host plant^15^. Some research, such as^16^ studied the impact of climate change on the climatic range of *Abies religiosa* (white pine), a preferred host for monarch butterflies during their winter migration in Mexico. The study predicts a significant decrease in the suitable area for *Abies religiosa* by the end of the century, which could impact the overwintering sites of monarch butterflies in Mexico and requires conservation strategies to protect them.

In our work we used statistical models to predict species distributions over a broader range, taking into account multiple environmental variables. Some authors^17^ have studied the monarch butterfly (*Danaus plexippus*)-*Asclepias* plant interaction under climate change. The research revealed that climate change can negatively affect the relationship between monarch butterflies and milkweed plants, potentially creating ecological traps that harm the survival and success of these butterflies. Previous studies examined the causes of monarch butterfly declines in eastern North America, focusing on three hypotheses: (1) Loss of milkweed plants due to increased herbicide use; (2) Mortality during fall migration and/or early winter establishment; and (3) Changes in breeding-season climate^18^. The research found that breeding-season weather plays a dominating role in determining monarch butterfly population sizes, with potential impacts on their breeding range due to climate change.

The amount of rainfall throughout the breeding season affects the vigour and number of plants that provide shelter and food for larval stages of the monarch butterflies^19^. Climate can also indirectly affect *D. plexippus* populations by impacting the availability of the host plant as the species larvae are known to be specialist herbivores, that feed exclusively on the milkweed plant (genus *Asclepias* spp). Studies show that higher temperatures deteriorate the quality of milk weed as the host plant, which adversely affects the development of monarch butterfly larvae that feed on them^20^. Temperature fluctuations during the breeding season can impact host plant availability and influence the development and survival rates of monarch larvae^21^.

The correlation between monarch butterfly life cycles and climatic conditions indicates that the species may be negatively impacted by the ongoing effects of global climate change^22^. When predicting changes in a species’ range under various climate change scenarios, species distribution models (SDMs) can be very helpful. These models use environmental variables to predict species’ spatial distribution, offering insights into how climate changes may impact their habitats^23^. SDMs identify areas providing adequate habitat for species survival and those areas more at risk due to changing climatic conditions^24^. Studies have employed SDMs to project the impact of climate change on *D. plexippus* populations, their breeding grounds, and host plants. Researchers^25^ projected major changes in the abundance of the North American monarch population because of the climate change impact on the timing of host plant growth (milk weed phenology) and reproductive success of monarch butterflies (productivity). Similarly, predicted changes in the distributions of monarch butterfly host plants (showy and swamp milkweed- *A. speciosa* and *A. incarnata*) revealed the role of environmental and climatic factors in influencing host plant distribution and, in turn, the spread of monarch populations^26^.

Species may move to more favourable climates in response to climate change, a process called range shifting^27^the degree to which this happens depends on how well a species can adapt to climate change^28^. Due to host plant specificity, global climate change poses a serious threat to the extinction of monarch butterflies.

Previous SDM-based studies on monarch butterfly distributions have advanced our understanding of climate-driven range changes, but they also have limitations^23–26^. Many rely exclusively on bioclimatic variables and omit important ecological predictors such as land cover and vegetation structure, which influence monarch breeding and migration by affecting host plant distribution. These models are also frequently applied at broad spatial scales without incorporating species-specific ecological traits, such as the monarch’s dependence on *Asclepias* species for larval development, which can reduce predictive accuracy in ecologically heterogeneous regions. As a result, earlier models may over- or under-predict suitable habitat in areas where climate alone does not capture habitat suitability dynamics.

This study contributes to the growing body of research on climate change impacts by applying species distribution models (SDMs) to project future habitat suitability for *D. plexippus* (monarch butterfly) across multiple emission scenarios. In addition to standard bioclimatic variables, we incorporate both altitude and land cover which are important additions given the monarch’s strong dependence on milkweed availability and suitable breeding habitats. Land cover change, including urbanization and agricultural expansion, also plays a key role in shaping monarch distribution, yet it is often underrepresented in large-scale modeling studies. By integrating land cover into SDMs, our work offers a more ecologically nuanced projection of potential habitat shifts, thereby informing region-specific conservation strategies.

The aims of this study are to: (i) assess the current global distribution range of *D. plexippus*, considering both climate and ecological factors; (ii) investigate potential impacts of climate variability on habitat suitability, focusing on temperature and varying precipitation patterns affecting *D. plexippus*; (iii) forecast the future distribution range of *D. plexippus* for the time periods of 2021–2041, 2041–2060 and 2081–2100 under different climate scenarios, and to compare these modeled projections; and (iv) examine which climatic variables and ecological determinants are, or are not, influencing the distribution of *D. plexippus.*

## Methods

### Occurrence records

**Fig. 1:**
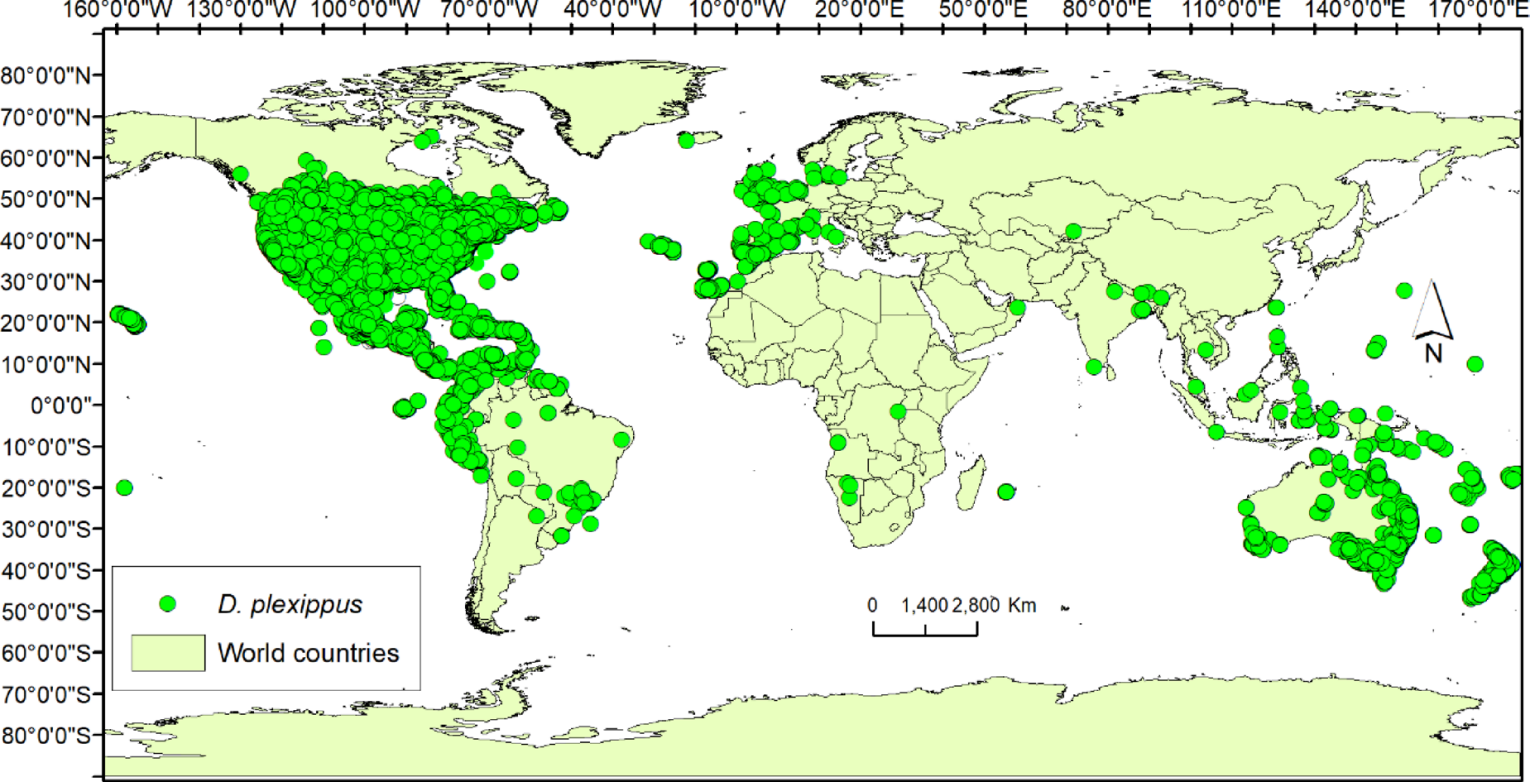
Global distribution of *D. plexippus* occurrence records obtained from GBIF^29^.

We retrieved geo-referenced records for *D. plexippus* from the Global Biodiversity Information Facility. The occurrence data totaled 583,561 entries that were a combination of human observations and records of preserved specimens^29^. The compiled records were verified and refined to remove errors. Only records collected between 1950 and 2023 were retained to reduce potential historical biases and to better align occurrence data with the baseline period of the environmental predictors (WorldClim 2.0 current climate layers). Older records were excluded because they may not accurately reflect present-day distributions, especially given the rapid climate-driven shifts reported for *D. plexippus*. Records with a reported coordinate uncertainty greater than 5 km were excluded. This threshold ensures that only georeferenced points with sufficient spatial precision are used, which is especially important for species distribution modelling at a regional to global scale. Duplicate points and points with missing coordinates were also removed. Records falling outside the known range (e.g., records placed in oceans or implausible locations due to digitization errors) were excluded. Occurrences outside the known ecological range (e.g., extreme latitudes or elevations inconsistent with monarch butterfly ecology) were checked and removed if unsupported by credible documentation. These steps help minimize sampling errors, geolocation inaccuracies, and temporal mismatches that could introduce bias into the species distribution models. The cleaning process resulted in a reduction of the occurrence records to 175,307 observation points. The distribution of the cleaned records was mapped using ArcGIS 10.3^30^ (Fig. 1).

### Environmental predictors

We utilized nineteen standard bioclimatic variables representing the current climate (1970–2000) and Altitude, obtained at a spatial resolution of 2.5 arc-minutes (~ 5 × 5 km at the equator), from the WorldClim high-resolution climate layers dataset^31^. These variables were employed to predict potential habitat suitability for *D. plexippus* on a global scale. The worldwide land cover distribution data were retrieved from https://earthexplorer.usgs.gov/ (accessed on June 13, 2023) in order to account for the changes in land cover. The environmental predictors were kept at 2.5 arc-minute spatial resolution across all spatial layers.

To avoid overfitting in the species distribution models (SDMs) and ensure that the selected predictors were statistically independent, we first examined all candidate environmental variables for multicollinearity^32^. This process involved calculating pairwise Pearson correlations and variance inflation factors (VIF) for the full set of 19 bioclimatic variables, plus land cover and altitude. Predictors with high pairwise correlation coefficients (*r* > 0.75) and VIF values greater than 5 were excluded to reduce redundancy and improve model interpretability. The VIF provides a robust measure of how much each predictor is explained by the others. Although several bioclimatic variables initially appeared relevant, they were strongly correlated with each other, which would have risked inflating the model’s variance and reducing generalizability. After careful screening, we retained three predictors, land cover, annual precipitation, and altitude, that met the correlation and VIF criteria and are also ecologically meaningful for *D. plexippus*. Land cover represents the availability of breeding and feeding habitat, annual precipitation affects host plant distribution and nectar availability, and altitude captures important topographic and climatic gradients.

Ensemble modeling was then performed using three widely used algorithms, generalized linear models (GLM), Random Forests (RF), and Boosted Regression Trees (BRT), implemented in the sdm package in R 4.2.0^33^. These algorithms have demonstrated good predictive performance, stability, and transferability for ecological applications^34^. We evaluated the model accuracy using the True Skill Statistic (TSS) and the area under the receiver-operating characteristic curve (ROC-AUC)^35^. TSS was used to assess the ensemble models, providing a measure that combines sensitivity and specificity. Unlike other metrics, TSS remains unaffected by prevalence, making it valuable for predicting species presence and absence reliably. This is a key factor in ecological research, where species distribution can vary markedly within their habitats and overall host ranges. By employing TSS, we ensure the model’s predictive power and validate the suitability of habitats under current and future climate scenarios. As advised by^36^ we applied the Maximum Training Sensitivity Plus Specificity (MTSS) criterion for threshold estimation. The best threshold for transforming continuous habitat suitability maps into binary presence/absence maps was determined using the MTSS. We created binary maps (presence/absence) based on the MTSS threshold from continuous maps of the suitability of the current and future habitats to illustrate changes in the habitat. The global maps presented in Figs. 1, 4, 6 and 7, and 8 were created using ArcGIS Desktop version 10.8 (Esri, Redlands, CA, USA; https://www.esri.com/en-us/arcgis/products/arcgis-desktop/overview).

### Model Building

The model was trained using roughly 70% of the occurrence data, with the remaining 30% being used for testing and validation^34^. Using the ‘sdm’ package and R 4.2.0, the generalized linear model (GLM) was built to simulate the distribution of *D. plexippus* and weighted by TSS^33^. The MTSS threshold rule was implemented^36^ and the models’ accuracy was evaluated using the TSS and AUC^35^.

### Risk assessment under climate scenarios

To forecast potential shifts in the distribution of *D. plexippus* under climate change, we used two downscaled global climate models (GCMs) from the WorldClim 2.0 database: the Beijing Climate Center - Climate System Model version 2 - Medium Resolution (BCC-CSM2-MR) and Institute Pierre-Simon Laplace - Climate Model version 6 A - Low Resolution (IPSL-CM6A-LR). These models are part of the Coupled Model Intercomparison Project Phase 6 (CMIP6), the latest international framework coordinated by the Intergovernmental Panel on Climate Change (IPCC) for its Sixth Assessment Report (AR6)^37^. CMIP6 provides state-of-the-art global climate projections under multiple scenarios (Shared Socioeconomic Pathways, SSPs) and is widely recognized as the current scientific standard for global and regional climate impact studies^38^.

For our projections, we applied two SSPs: the high-emission Shared Socioeconomic Pathway 5 (SSP5-8.5) scenario and the low-emission, Shared Socioeconomic Pathway 1 – Representative Concentration Pathway 2.6 (SSP1-2.6) scenario. Predictions were made for the near future (2021–2040) and the far future (2041–2060 and 2081–2100). For each period, we estimated changes in the potential species distribution compared to current conditions.

Quantitative maps produced by simulating the distribution of species under present and projected climatic conditions were transformed into binary suitability maps based on the MTSS threshold in order to evaluate the risk of habitat loss and changes in species distribution. The ArcGIS 10.3 framework was utilized to process all of the maps and spatial layers^30^. This all-encompassing strategy guarantees a careful assessment of the possible effects of climate change on *D. plexippus* habitat and distribution.

**Table 1:** List of predictor variables used to model the distribution of *D. Plexippus.*

Variable short name	Variable long name	Units
Alt	Altitude	m
Bio1	Annual Mean Temperature	°C
Bio2	Mean Diurnal Range (Mean of monthly (max temp - min temp))	°C
Bio3	Isothermality (P2/P7) (* 100)	--
Bio4	Temperature Seasonality (standard deviation *100)	--
Bio5	Max Temperature of Warmest Month	°C
Bio6	Min Temperature of Coldest Month	°C
Bio7	Temperature Annual Range (P5-P6)	°C
Bio8	Mean Temperature of Wettest Quarter	°C
Bio9	Mean Temperature of Driest Quarter	°C
Bio10	Mean Temperature of Warmest Quarter	°C
Bio11	Mean Temperature of Coldest Quarter	°C
Bio12	Annual Precipitation	mm
Bio13	Precipitation of Wettest Month	mm
Bio14	Precipitation of Driest Month	mm
Bio15	Precipitation Seasonality (Coefficient of Variation)	--
Bio16	Precipitation of Wettest Quarter	mm
Bio17	Precipitation of Driest Quarter	mm
Bio18	Precipitation of Warmest Quarter	mm
Bio19	Precipitation of Coldest Quarter	mm
Land cover	Land cover	m^2^

## Results

### Model performance and potential response to bioclimatic variables

**Fig. 2:**
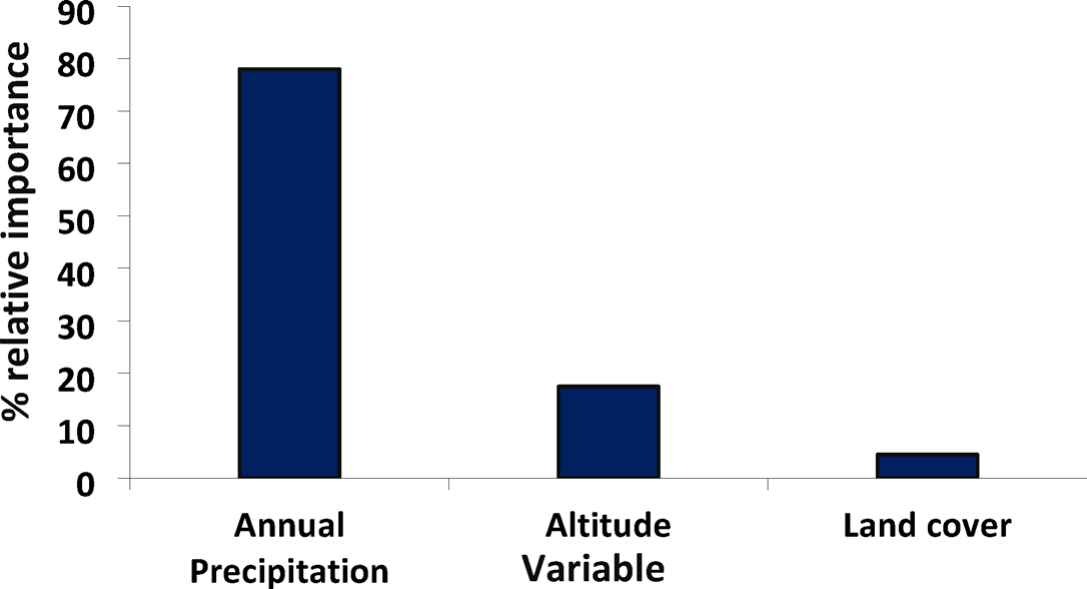
Ranking of factors in predicting the distribution of *D. plexippus* using the percentage relative importance.

**Fig. 3:**
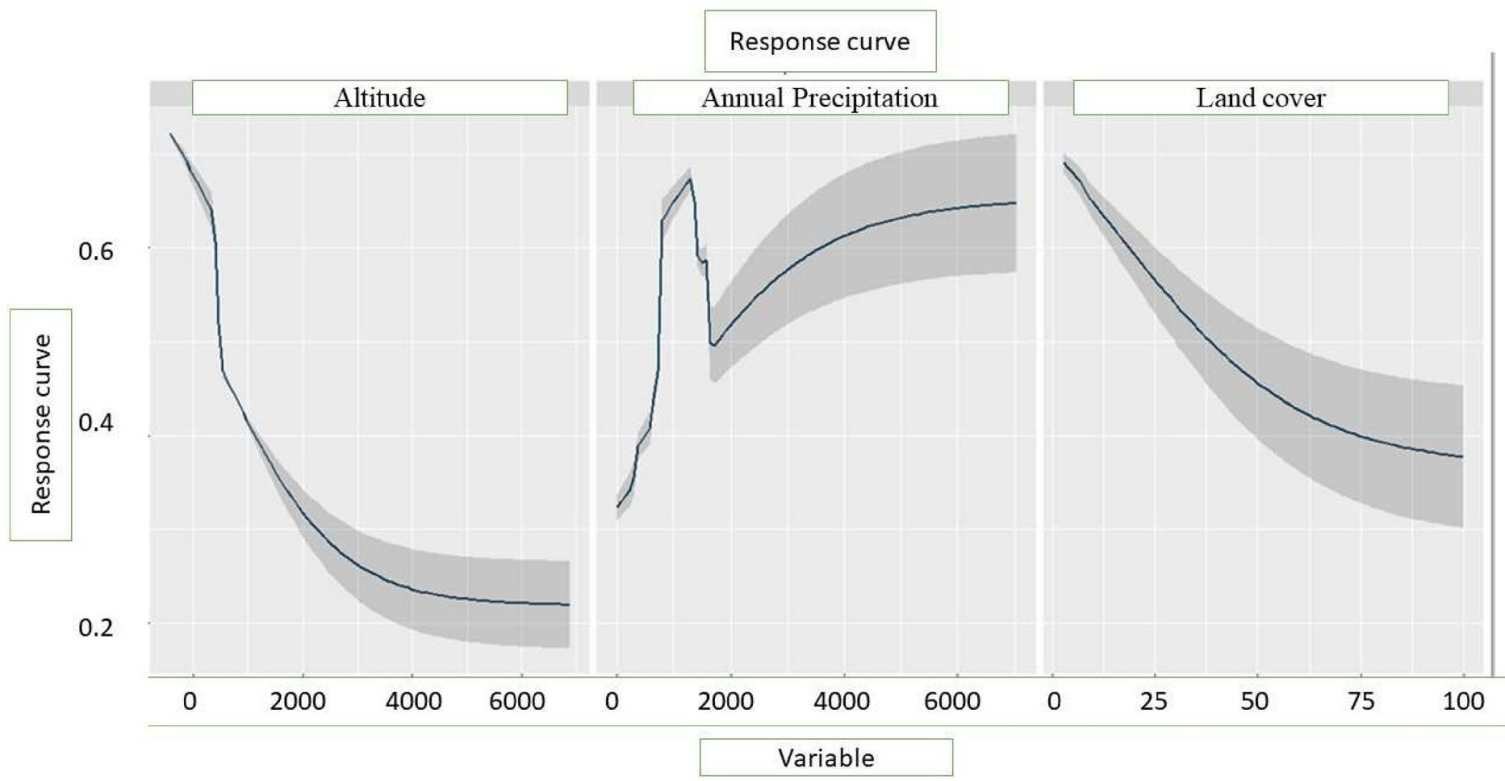
Response curves for the key variables used in the distribution modeling of *D. plexippus.*

Table 1 lists the uncorrelated variables that were used in the modeling. With an Area Under the Curve (AUC) of 0.75 and a TSS of 0.52, the generalized linear model (GLM) demonstrated excellent performance and great accuracy (Table 2). With a relative value ranging from more than 4.5–78%, the annual precipitation (Bio12), altitude, and type of land cover were shown to be the most significant variables in determining the probable distribution of monarch butterflies (Fig. 2; Table 3). As the chance of occurrence of the species increased with an increase in annual precipitation beyond 1,000 mm, the response curves showed that the species preferred locations with higher precipitation (Bio12) (Fig. 3). Additionally, it was shown that species do not favor higher elevations because their likelihood of occurring dropped as altitude (Alt) increased above 2,000 m.

**Fig. 4:**
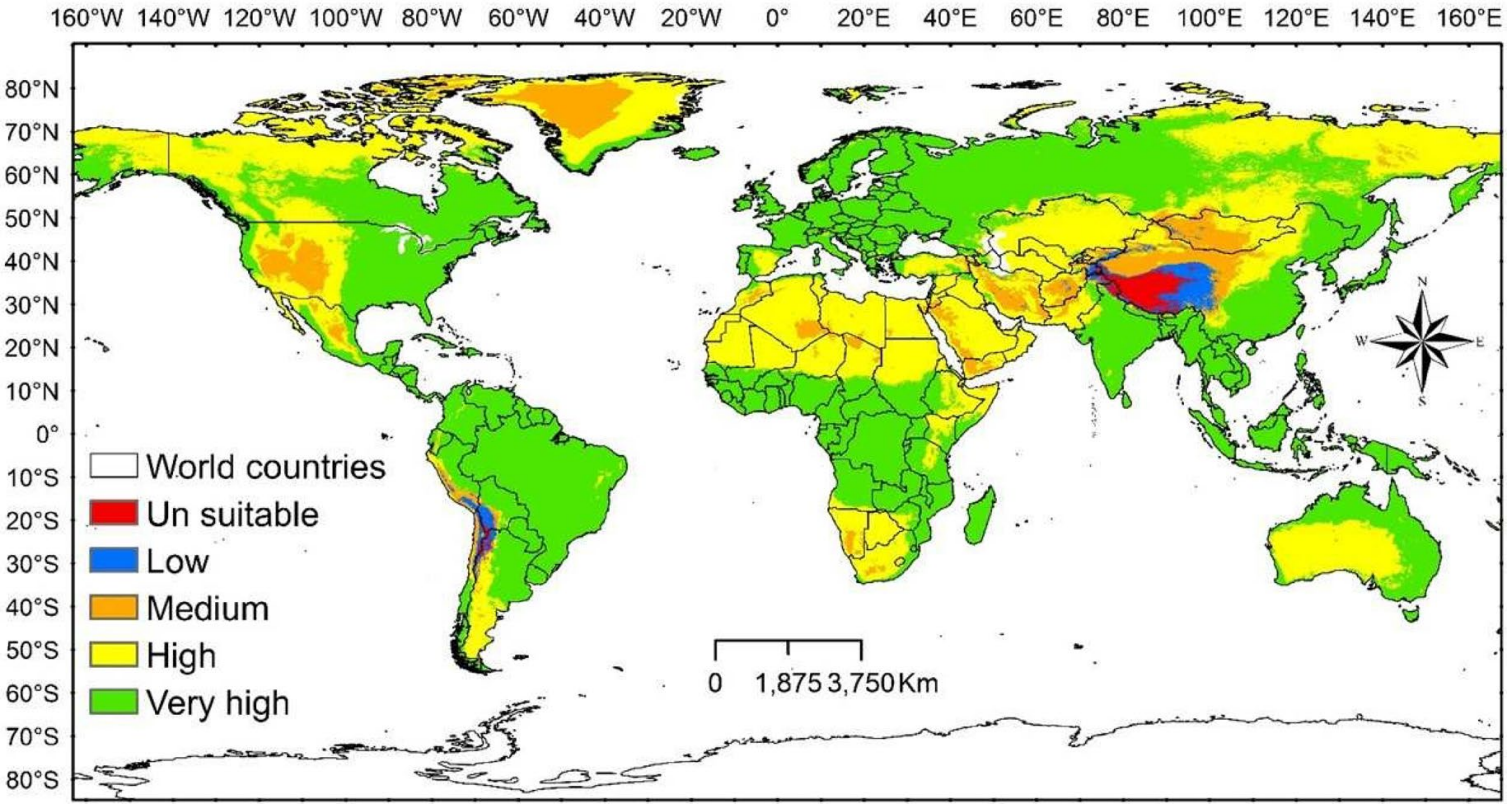
The potential distribution of the suitability categories under current climate conditions range of *D. plexippus.*

Temperate habitats were the most appropriate land cover categories for the distribution of *D. plexippus*. The species prefers temperate and tropical humid regions, with highly suitable areas concentrated on the eastern side of the Americas, northern Europe, sub-Saharan Africa, East Asia, and Australia, according to the species’ potential distribution under the current climate conditions. The western edge of the Americas, North Africa, and Central and West Asia contained the majority of the most inappropriate regions (Fig. 4).

**Table 2:** Four methods: GLM, GAM, BRT and RF analyzed for the area under the curve (AUC), the true skill statistic (TSS) and deviance values for predicting modeled distribution of *D. Plexippus.*

Methods	AUC	TSS	Deviance
Generalized linear model (GLM)	0.75	0.52	1.26
Generalized Additive Model (GAM)	0.82	0.64	0.88
Boosted Regression Trees (BRT)	0.81	0.65	0.99
Random Forest (RF)	0.97	0.85	0.33

**Table 3:** Percent contribution and the value of the variance inflation factor (VIF) for the variables in the model predicting distribution of *D. Plexippus.*

Variables	Units	Percent (%)	VIF
Altitude	m	17.5	1.31
Annual Precipitation	mm	78	1.30
Land cover	m^2^	4.5	1.04

### Current and projected suitability under climate change scenarios

The potential *D. plexippus* distribution under future climate scenario based on the ssp1 2.6 and ssp5 8.5 scenarios of two general climate scenarios, BCC-CSM2MR and IPSL-CM6ALR, projected for the near future (2021–2040), and the far future (2041–2060 and 2081–2100) are illustrated in Figs. 6, 7 and 8.

Overall, across all scenarios considered in the two global climate models, it is evident that the loss in potential suitable habitat observed under current climate conditions will continue over time, with far-future projections indicating a more substantial loss compared to near-future estimates. Specifically, the potential habitat loss under low-emission scenario (SSP1 2.6) is notably high in the BCC-CSM2-MR model compared to the model of IPSL-CM6A-LR. Conversely, under high-emission scenario (SSP5 8.5), the model of IPSL-CM6A-LR predicts a higher loss in potential habitats, especially in far-future projections (refer to Table 4; Figs. 6, 7 and 8).

However, irrespective of the emission scenarios, both low and high, the stable area is projected to decrease in far-future scenario compared to near-future projections (Table 4). It is noteworthy that under the high-emission scenario of the BCC-CSM2-MR model, there is a prediction of greater habitat gain compared to the low-emission scenario. In general, across all scenarios investigated in the two global climate models, the gain area is predominantly observed in central and northern Asia, central and eastern Africa, northern Europe, and central and northern parts of North America (Figs. 6, 7 and 8). These findings suggest that the species is likely to undergo a northward latitudinal shift in addition to an altitudinal shift.

### The predicted future distribution

**Fig. 5:**
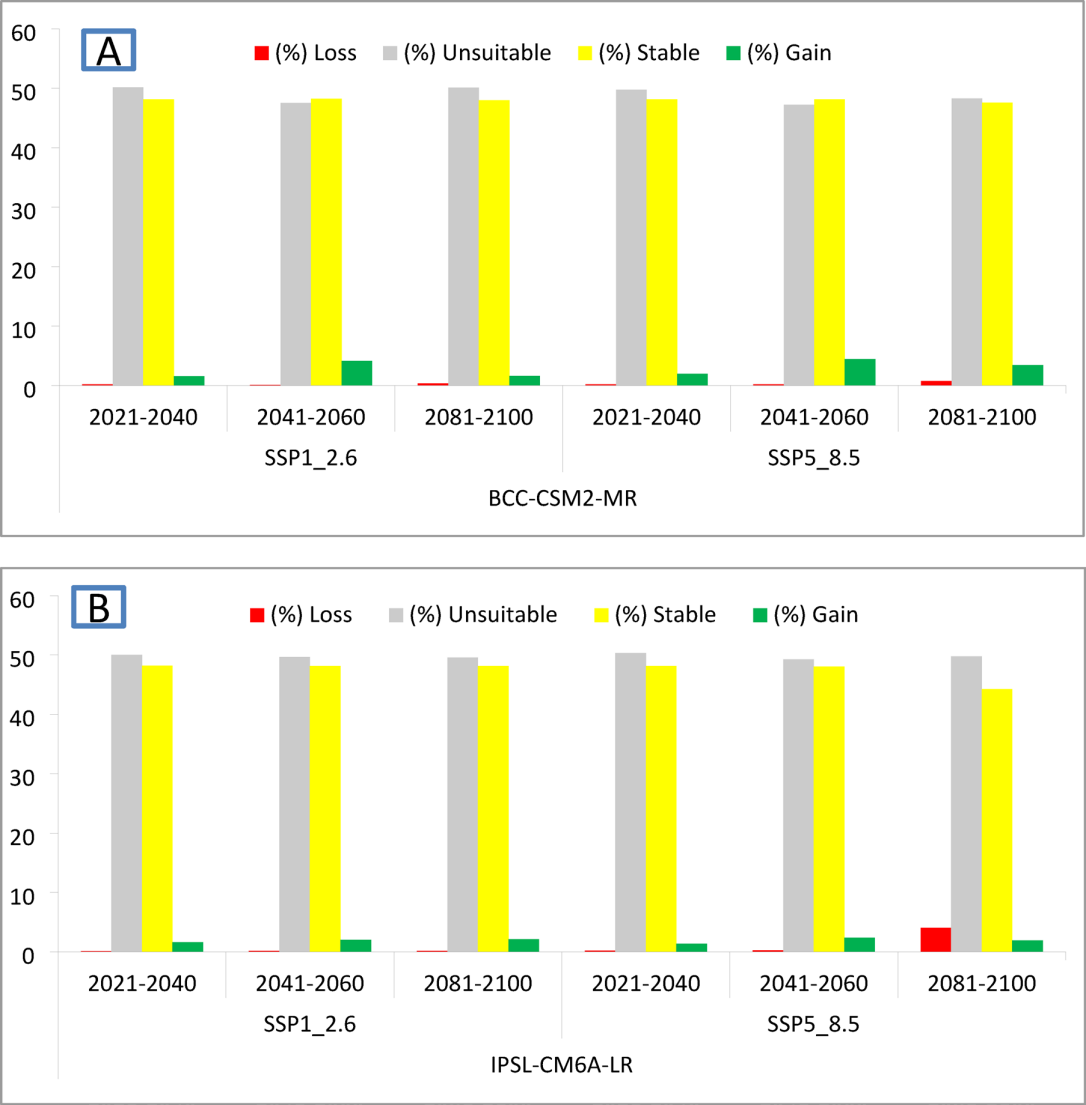
Panel **(A)** The percentages of predicted loss, gain, unsuitable, and stable habitat areas for *D. plexippus* future scenarios, SPP1_2.6 and SSP5_8.5, of BCC-CSM2MR for the years 2021–2040, 204–2060 and 2081–2100. Panel **(B)** The percentages of predicted loss, gain, unsuitable, and stable habitat areas for *D. plexippus* future scenarios, SPP1_2.6 and SSP5_8.5, of IPSL-CM6ALR, for the years 2021–2040, 204–2060 and 2081–2100.

Under the low emission scenario ssp126 of the BCC-CSM2-MR climate model, the species is projected to experience habitat losses of 0.21%, 0.11%, and 0.35%, and habitat gains of 1.54%, 4.15%, and 1.59% during the years (2021–2040), (2041–2060), and (2081–2100), respectively. Similarly, under the low emission scenario of the model of IPSL-CM6A-LR, there is a forecasted loss in habitat area of 0.13%, 0.16%, and 0.16%, and a gain of 1.65%, 2.01%, and 2.14% during the corresponding years (Table 4; Fig. 5).

In high emission scenarios of climate model BCC-CSM2-MR, *D. plexippus* is anticipated to undergo habitat losses of 0.18%, 0.21%, and 0.77%, and habitat gains of 1.97%, 4.46%, and 3.41% in the years (2021–2040), (2041–2060), and (2081–2100), respectively. Similarly, under the high emission scenario of climate model IPSL-CM6A-LR, a loss in habitat area of 0.20%, 0.27%, and 4.05%, and a gain of 1.38%, 2.41%, and 1.94% in *D. plexippus* habitat is projected for the same years (refer to Table 4; Fig. 5).

Although the predicted percentages of habitat loss or gain appear low (0.11–4.05%), they translate to considerable absolute areas given the large baseline habitat extent (~ 2.75 million km²). For example, a 0.11% loss equates to about 3,025 km², while a 4.05% gain represents over 111,000 km² of additional suitable habitat.

In high emission scenarios of the BCC-CSM2-MR climate model, a relatively greater loss is anticipated, particularly in the far future. However, it also projects more gain in some areas compared to low emission scenarios. Conversely, under high emission scenarios (SSP5-8.5), the IPSL-CM6A-LR models project a higher potential habitat loss for *D. plexippus*, while the BCC-CSM2-MR model indicates a greater potential habitat gain (Table 4).

When comparing the two models, BCC-CSM2-MR generally projects more loss than IPSL-CM6A-LR under low emission scenarios, with comparable or higher habitat gain in the mid-century projection. On the other hand, under high emission scenarios, IPSL-CM6A-LR forecasts more loss and less gain than BCC-CSM2-MR.

**Table 4:** The percentage predicted loss, gain, stable and unsuitable area of *D. plexippus* under three future scenarios, SPP1_2.6 and SSP5_8.5, of two general climate scenarios, BCC-CSM2MR and IPSL-CM6ALR, for the years 2021–2040, 2041–2060 and 2081–2100 compared to the current status.

GCM	Scenario	Period	(%)			
			Loss	Unsuitable	Stable	Gain
BCC-CSM2-MR	SSP1_2.6	2021–2040	0.21	50.15	48.11	1.54
		2041–2060	0.11	47.54	48.21	4.15
		2081–2100	0.35	50.09	47.96	1.59
	SSP5_8.5	2021–2040	0.18	49.72	48.13	1.97
		2041–2060	0.21	47.23	48.11	4.46
		2081–2100	0.77	48.27	47.55	3.41
IPSL-CM6A-LR	SSP1_2.6	2021–2040	0.13	50.03	48.19	1.65
		2041–2060	0.16	49.68	48.15	2.01
		2081–2100	0.16	49.54	48.16	2.14
	SSP5_8.5	2021–2040	0.20	50.31	48.12	1.38
		2041–2060	0.27	49.27	48.04	2.41
		2081–2100	4.05	49.74	44.27	1.94

**Fig. 6:**
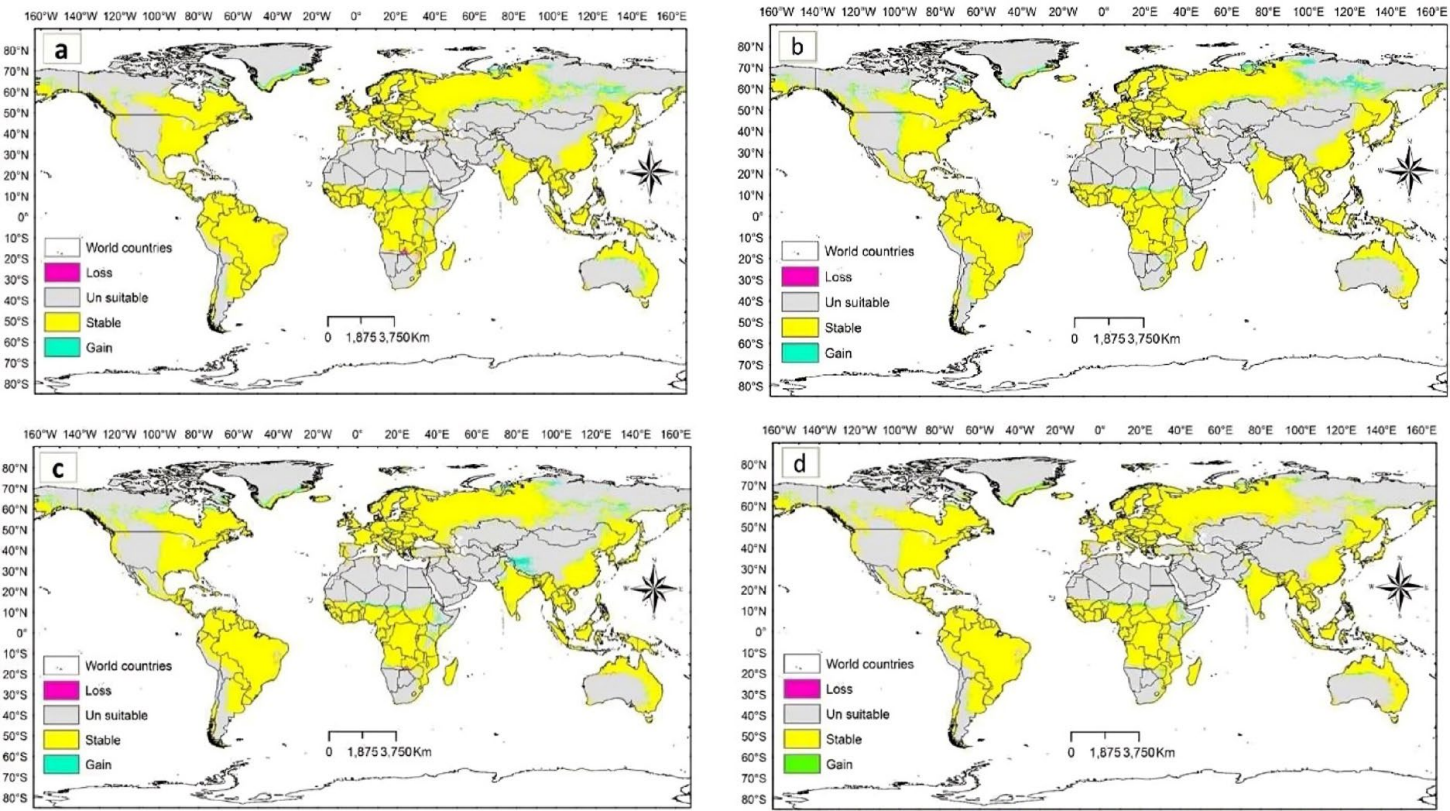
Gain and loss in suitable habitats of *D. plexippus* projected for the period 2021–2040 under the two future scenarios **(a)**BCC-CSM2MR_ssp126 **(b)** BCC-CSM2MR_ssp585 **(c)** IPSL-CM6ALR_ssp126 and **(d)** IPSL-CM6ALR_ssp585.

**Fig. 7:**
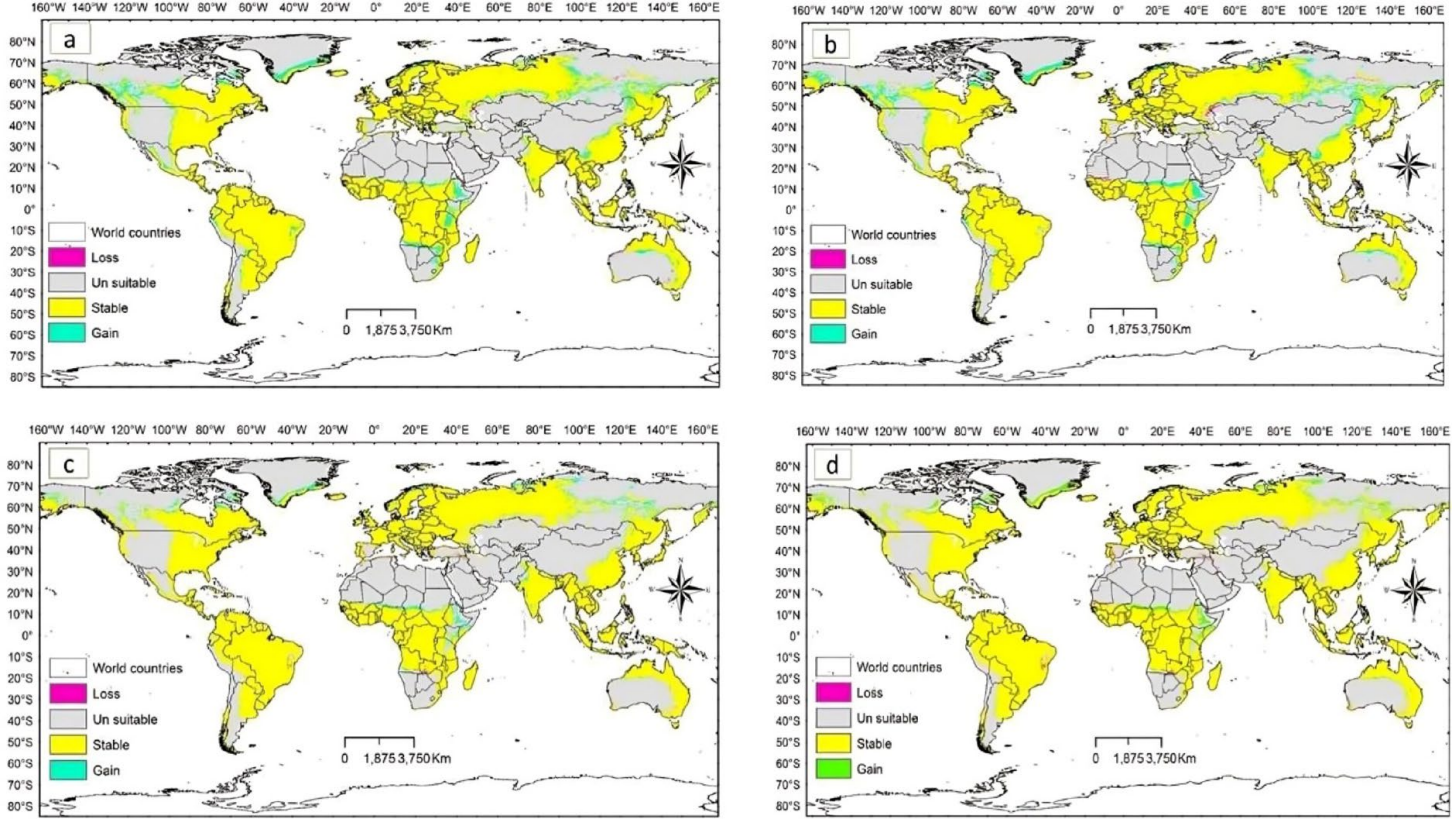
Gain and loss in suitable habitats of *D. plexippus* projected for the period 2041-2060 under the two future scenarios **(a)** BCC-CSM2MR_ssp126 **(b)** BCC-CSM2MR_ssp585 **(c)** IPSL-CM6ALR_ssp126 and **(d)** IPSL-CM6ALR_ssp585.

**Fig. 8:**
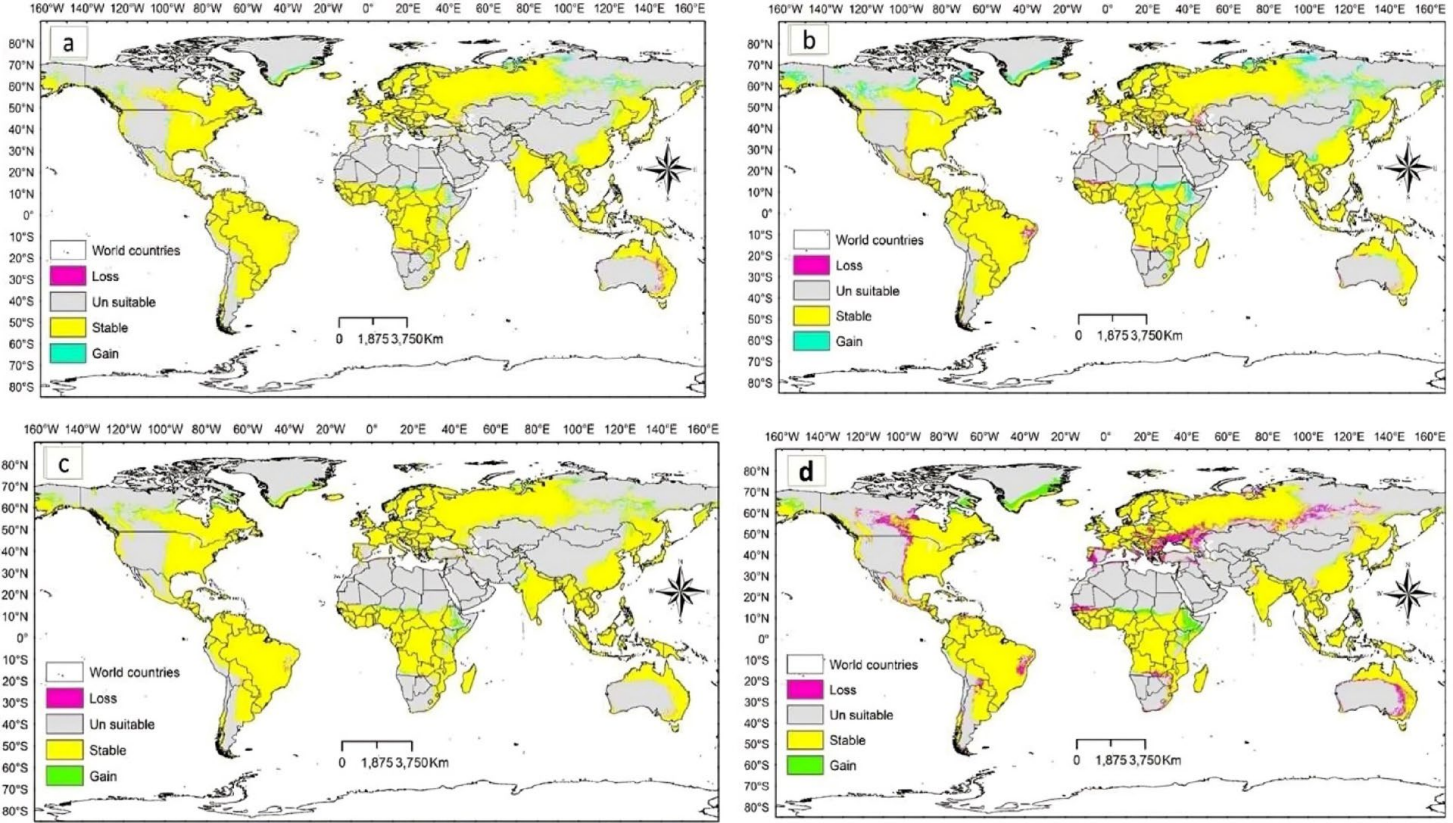
Gain and loss in suitable habitats of *D. plexippus* projected for the period 2081-2100 under the two future scenarios **(a)** BCC-CSM2MR_ssp126 **(b)** BCC-CSM2MR_ssp585 **(c)** IPSL-CM6ALR_ssp126 and **(b)** IPSL-CM6ALR_ssp585.

The habitat suitability maps (Figs. 6, 7 and 8) visually highlight areas where habitat gain is predicted, but these gains are not uniformly distributed across all regions. When we look at the absolute area of habitat gain and loss by region (Table 5), it becomes clear that many areas, especially in Africa, Asia, and South America are projected to experience significant net losses in suitable habitat under both low- and high-emission scenarios. Meanwhile, other regions such as North America and Europe may see localized gains, particularly under near-future scenarios. This explains the apparent discrepancy between the mapped visual outputs and the numerical summaries: Figures 6, 7 and 8 are binary presence/absence maps based on our SDM projections and highlight areas where new suitable habitat appears (habitat gain), but the overall balance of habitat change reflects regional and global losses offset by other areas. Even though the regional trend may show a small net change (loss) or near balance, some regions are projected to face considerable contractions in suitable habitat (especially for Africa, South America and Asia in far-field projections).

**Table 5:** Summary table that contrasts profits and losses in some regions.

Scenarios	Region	Total Area (Mkm^2^)	Loss (Mkm²)	Gain (Mkm²)	Net Change (Mkm²)
BCC-CSM2-MR_ssp126_ 2021–2040	North America	24.7	3.9	5.9	+ 2.0
	Europe	10.2	0.8	1.5	+ 0.7
	Asia	44.6	11.2	10.0	−1.2
	South America	17.8	6.2	2.1	−4.1
	Africa	30.4	8.5	4.3	−4.2
BCC-CSM2-MR_ssp126_ 2041–2060	North America	24.7	4.9	4.9	0.0
	Europe	10.2	1.0	1.0	0.0
	Asia	44.6	13.4	8.9	−4.5
	South America	17.8	7.1	1.8	−5.3
	Africa	30.4	9.1	3.0	−6.1
BCC-SM2-MR_ssp126_ 2080–2100	North America	24.7	5.2	4.2	−1.0
	Europe	10.2	1.2	1.0	−0.2
	Asia	44.6	12.5	9.4	−3.1
	South America	17.8	6.9	1.6	−5.3
	Africa	30.4	9.7	3.3	−6.4
BCCSM2_ MR_ssp585_ 2021–2040	North America	24.7	4.5	5.2	+ 0.7
	Europe	10.2	0.9	1.3	+ 0.4
	Asia	44.6	11.6	10.2	−1.4
	South America	17.8	6.1	2.0	−4.1
	Africa	30.4	8.8	4.3	−4.5
BCCSM2_ MR_ssp585_ 2041–2060	North America	24.7	5.9	3.9	−2.0
	Europe	10.2	1.5	0.8	−0.7
	Asia	44.6	15.6	6.7	−8.9
	South America	17.8	8.0	1.2	−6.8
	Africa	30.4	10.7	2.1	−8.6
BCC-SM2-MR_ssp585_ 2080–2100	North America	24.7	5.7	4.0	−1.7
	Europe	10.2	1.4	0.9	−0.5
	Asia	44.6	14.2	7.1	−7.1
	South America	17.8	7.8	1.3	−6.5
	Africa	30.4	10.4	2.7	−7.7
IPSL-CM6A_ LR_ssp126_ 2021–2040	North America	24.7	6.4	3.5	−2.9
	Europe	10.2	1.7	0.7	−1.0
	Asia	44.6	16.8	5.3	−11.5
	South America	17.8	8.7	0.9	−7.8
	Africa	30.4	12.5	1.6	−10.9
IPSL-CM6A_ LR_ssp126_ 2041–2060	North America	24.7	6.2	3.7	−2.5
	Europe	10.2	1.8	0.6	−1.2
	Asia	44.6	17.8	4.5	−13.3
	South America	17.8	8.9	0.9	−8.0
	Africa	30.4	12.2	1.5	−10.7
IPSL-CM6A- LR_ssp126_ 2080–2100	North America	24.7	7.2	2.8	−4.4
	Europe	10.2	2.1	0.5	−1.6
	Asia	44.6	17.8	4.5	−13.3
	South America	17.8	9.3	0.7	−8.6
	Africa	30.4	13.4	1.2	−12.2
IPSL-CM6A_ LR_ssp585_ 2021–2040	North America	24.7	6.9	3.0	−3.9
	Europe	10.2	1.8	0.6	−1.2
	Asia	44.6	17.8	4.5	−13.3
	South America	17.8	9.2	0.7	−8.5
	Africa	30.4	13.4	1.2	−12.2
IPSL-CM6A_ LR_ssp585_ 2041–2060	North America	24.7	6.7	3.2	−3.5
	Europe	10.2	1.8	0.7	−1.1
	Asia	44.6	15.6	5.8	−9.8
	South America	17.8	8.4	1.0	−7.4
	Africa	30.4	11.4	1.8	−9.6
IPSL-CM6A-LR_ssp585_ 2080–2100	North America	24.7	7.4	2.5	−4.9
	Europe	10.2	2.0	0.5	−1.5
	Asia	44.6	17.8	4.5	−13.3
	South America	17.8	9.6	0.7	−8.9
	Africa	30.4	13.4	1.2	−12.2

Table 5 compares all scenarios, showing estimated land changes by region in million square kilometers (Mkm²) and percentages in details. Our findings emphasize that conservation responses should be region-specific. While new habitat may emerge in higher latitudes, it does not guarantee connectivity or suitability for monarch populations without coordinated habitat corridors, host plant availability, and protection from non-climatic stressors.

## Discussion

### Effect of environmental variables on *D. plexippus* distributions

Distribution of *D. plexippus* is notably influenced by key bioclimatic variables, with altitude (Alt), land cover, and annual precipitation (Bio12) emerging as the most significant factors. These findings align with previous studies that similarly highlight the impact of rainfall on the quantity and quality of plants that provide shelter and food during the monarch butterflies’ larval stages^20^. The identified variables, namely altitude, land cover, and annual precipitation, are likely to play an important role in shaping distribution patterns of *D. plexippus*. The potential distribution of *D. plexippus* under future climate change conditions, as determined by our model reinforces the importance of these variables. The use of a minimal, non-redundant uncorrelated predictor set, together with cross-validated TSS and AUC metrics, reduces the likelihood of overfitting. However, employing additional sensitivity tests could further support models robustness and should be considered in future research.

### Effect of climate conditions on habitat suitability for *D. plexippus*

The outcomes from the models reveal a complex potential pattern for *D. plexippus*, as there are projections of both losses and gains in suitable habitat areas across different geographical regions. This nuanced response can be attributed to intricate interplay of climate patterns, which may either favor or impede species distribution through dynamic and region-specific seasonal interactions, leading to losses in the suitable habitats in some regions and gains in others, as highlighted by studies such as^39^. The species’ preferences for warm winters and spring, as documented in studies by^40^ become evident. However, the harmful effects of extremely hot, dry, cold, or wet environments on monarch populations are equally significant, as observed in studies by^25^. This delicate balance underscores the need for a comprehensive understanding of climate-induced impacts on *D. plexippus* and emphasizes the importance of tailored conservation strategies to address the diverse challenges posed by changing environmental conditions.

### Limitation of species distribution models (SDMs)

SDMs are valuable for projecting species distributions under different climate scenarios. They focus on climatic shifts and broad-scale environmental predictors. However, a limitation of SDMs is their inability to account for other important ecological and anthropogenic stressors that can greatly influence species distributions. For example, pesticides, invasive species, and land use change or degradation of insect host plants are not usually captured in SDMs. It is also important to recognize that land use changes such as urbanization, habitat fragmentation, and infrastructure development can significantly affect habitat suitability. These anthropogenic factors are not usually incorporated into SDMs due to data limitations, particularly at global scales and in lower-income countries (LICs) or regions affected by conflict (civil wars) where fine-resolution land use or chemical exposure data are unavailable or inconsistent. As a result, our projections, like those of many SDM-based reports, should be interpreted with the context of identifying broad climatic influences rather than definitive species ranges. Future research could incorporate finer-scale ecological data or couple SDMs with mechanistic or hybrid modeling approaches to enhance accuracy. Despite the limitations, climate-driven trends identified in our analysis provide a useful framework for identifying areas of concern for conservation efforts and prioritizing adaptive management responses.

### Conservation implications for *D. plexippus*

Across all scenarios explored in the two global climate models, the predominant gain of habitat areas for *D. plexippus* were identified in central and north Asia, central and eastern Africa, northern Europe, and central and northern parts of North America. This observation suggests a notable northern latitudinal shift, complementing the anticipated altitudinal shift. This aligns with findings from other studies, such as^41^ that predicted a substantial distribution shift of the monarch butterfly overwintering groves in California under climate change scenarios, including a local altitudinal shift. The study also revealed a local altitudinal shift with the occurrence probability of overwintering habitat decreasing in the low elevation coastal, while it is increasing in higher elevation regions. The study revealed continuation of this trend under the higher emission scenarios, where the most suitable areas will be restricted along ridges and mountain tops in the region.

The model outputs have significant conservation implications for *D. plexippus*. Potential management strategies include a focus on habitat restoration, prioritization of overwintering sites, and adaptive management approaches. In Mexico, specifically within the monarch Butterfly Biosphere Reserve in Michoacán, three UNESCO-designated World Heritage Sites-Cerro Altamirano, Chincua-Campanario-Chivati-Huacal, and Cerro Pélon-underscore the importance of conservation efforts^42^.

While not all overwintering sites have received formal UNESCO recognition, numerous countries and organizations are actively involved in conservation efforts globally. For instance, in California, coastal areas serve as monarch overwintering grounds, and the Xerces Society for Invertebrate Conservation leads the monarch Butterfly Conservation Program, engaging in research, habitat restoration, and community initiatives^43^.

In Africa, overwintering sites in Tanzania and South Africa prompt conservation initiatives focus on habitat restoration and community-based projects. Monarch butterfly conservation initiatives in South Africa are actively supported by groups such as the Endangered Wildlife Trust (EWT) and the South African National Biodiversity Institute (SANBI)^44^.

Similarly, in Europe, countries like Spain and Portugal host monarch butterfly overwintering sites, driving conservation measures to protect habitats and address threats like habitat fragmentation and climate change. Although dedicated monarch butterfly conservation programs may be lacking, broader biodiversity conservation initiatives, like those led by the Spanish Ornithological Society (SEO/BirdLife) and regional environmental agencies, indirectly benefit a range of species, including butterflies^45^.

## Conclusion

In conclusion, our study provides a better understanding of how global change may impact upon *D. plexippus* distribution in the world. The anticipated losses and gains in suitable habitats under diverse emission scenarios will affect the monarch’s adaptive response to changing environmental conditions. As long-term observational data from citizen science programs (e.g., Journey North: https://journeynorth.org/; and Monarch Watch: https://www.monarchwatch.org/) continue to accumulate, future studies will be able to evaluate whether projected shifts in habitat suitability, such as our modeled outputs for 2081–2100, are reflecting real-world changes in monarch butterfly host range and behavior.

These projected impacts unveil nuanced shifts in habitat suitability across migratory routes and host range, manifesting as a delicate balance of losses and gains across diverse global regions. In light of these findings, it is imperative for *D. plexippus* conservation initiatives to strategically prioritize regions confronted with imminent habitat loss. This urgency includes formulating forward-looking plans tailored to effectively mitigate the adverse effects of climate change on migratory monarch butterfly populations. A proactive focus on at-risk areas will help increase the resilience and sustainability for these iconic butterflies amidst ongoing environmental challenges.

Furthermore, these findings emphasize the intricate interplay between bioclimatic factors and the distribution dynamics of *D. plexippus*. This highlights the need for focused conservation efforts and adaptive management strategies to effectively address and reduce the impacts of global change on migratory species. Adopting and including proactive measures will ensure the continued well-being of *D. plexippus* populations in the face of future environmental changes.

## Data Availability

The occurrence data for *D. plexippu*s were obtained from GBIF. Bioclimatic variables of the climate were obtained from the WorldClim. Data supporting the findings of this study are available from the corresponding author upon reasonable request. Please contact Dr. Sanad H. Ragab, Department of Zoology and Entomology, Faculty of Science, Al-Azhar University, Nasr City, Cairo, Egypt (email: sanadragab@azhar.edu.eg).
